# Differentiation of the retinal morphology aging trajectories in schizophrenia and their associations with cognitive dysfunctions

**DOI:** 10.3389/fpsyt.2023.1207608

**Published:** 2023-07-19

**Authors:** Adam Domagała, Lucyna Domagała, Natalia Kopiś-Posiej, Michał Harciarek, Paweł Krukow

**Affiliations:** ^1^Department of Clinical Neuropsychiatry, Medical University of Lublin, Lublin, Poland; ^2^Non-Public Health Facility “OKO-MED”, Sandomierz, Sandomierz County, Poland; ^3^Department of Neuropsychology, Institute of Psychology, University of Gdańsk, Gdansk, Poland

**Keywords:** schizophrenia, retina, processing speed, accelerated aging, optical coherance tomography

## Abstract

Previous studies evaluating the morphology of the selected retinal layers in schizophrenia showed abnormalities regarding macular thickness, retinal nerve fiber layer (RNLF), and ganglion cell complex (GCC). Concurrently, accumulating neuroimaging results suggest that structural alterations of the brain in this disease might be an effect of accelerated aging. Referring to these findings, we aimed to determine whether the thinning of the retinal layers assessed with the optic coherence tomography (OCT) in a group of schizophrenia patients (*n* = 60) presents a significant age-related decrease exceeding potential changes noted in the control group (*n* = 61). Samples of patients and controls were divided into three age subgroups, namely, younger, middle-aged, and older participants. OCT outcomes, such as macular thickness and volume, macular RNFL, peripapillary RNFL, and GCC, were analyzed concerning a diagnosis status (controls vs. patients) and age subgroups. Additionally, associations between retinal parameters, age, and selected cognitive functions were evaluated. *post-hoc* tests revealed that macular thickness and volume in patients undergo significant age-dependent thinning, which was not observed in the control group. Regression analyses confirmed the association between macular morphology and age. Selected speed-dependent cognitive functions in patients decreased significantly with age, and these features were also significantly associated with some OCT outcomes also after controlling for antipsychotic treatment. Our results suggest that reduced measures of retinal structure detected in schizophrenia may be an effect of accelerated aging; however, further research is needed using computational solutions derived from brain imaging studies based on large datasets covering representatives of all age groups.

## Introduction

For approximately 20 years, there has been an increasing scope of research evaluating the condition of the retina in neuropsychiatric populations (1). The retina is an element of the central nervous system that can be directly observed and measured with precision analogous to histological sections (2). This anatomical structure originates from the neuroectoderm of the forebrain and is not myelinated. Additionally, the morphological complexity of the retina resembles, to some extent, the cortex with cell bodies, dendrites, and various neurotransmitter systems (3). The assessment of retinal morphology is usually performed with optical coherence tomography (OCT), enabling the measurement of various layers, including retinal nerve fiber layer (RNFL), macular volume and thickness (MV, MT), ganglion cell complex (GCC) containing cells, and inner plexiform layer (IPL) (4, 5). Accumulation of empirical reports documenting retinal morphology abnormalities in schizophrenia has contributed to several available meta-analyses covering these results. For example, Kazakos and Karageorgiou (6) confirmed significantly reduced thickness of peripapillary RNFL in schizophrenia patients, based on the results from 11 case-control studies. Lizano et al.'s (7) study based on measurements from 820 patients and 904 healthy subjects presented similar results, and GCC and IPL were also significantly thinner in patients than in controls. Komatsu et al. (8), after reviewing 23 cross-sectional studies, concluded that schizophrenia patients have significantly altered peripapillary RNFL, MV, MT, GCL-IPL, and the optic cup was compared with controls. In addition to confirming the morphological abnormalities in schizophrenia, meta-analyses' conclusions point to the necessity to incorporate larger groups of patients and controls for the potential impact of somatic factors on the retina condition in schizophrenia (9–11). Furthermore, a more thorough analysis revealed that retinal parameters decrease with the duration of illness (8, 12–14).

Currently, a growing number of studies have demonstrated that changes in the structure and function of the brain observed in schizophrenia may be explained by accelerated aging (15, 16). It is assumed that a substantial part of neuronal abnormalities observed in schizophrenia is dependent on a faster onset of pathophysiological processes typical of more advanced age than chronological age. In this perspective, structural brain changes, for example, thinning of selected cortical areas present in schizophrenia (15), may be the result of patients overtaking healthy subjects in the aging process comprising evaluated cortical regions. Several neuroimaging findings corroborated the existence of the “brain age gap” in this population, indicating that the aging of neuronal structures occurs faster than what would be expected considering the chronological age of patients (16, 17). Accelerated aging is also associated with the specificity of cognitive dysfunctions present in schizophrenia (18). Competences expressing substantial age dependence in the general population undergo a significant decline in schizophrenia, for example, processing speed, while factors associated with crystal intelligence, such as word recognition, which does not show negative age changes, are usually spared in schizophrenia.

In general, we assumed that retinal abnormalities in schizophrenia may arise from altered aging trajectories regarding this organ. Noting differences between patients and controls in OCT results only after taking into account patients with a longer duration of illness may be due to the fact that, in schizophrenia, the retina may start to age faster from a certain age or an illness duration threshold. Considering the above differences, we aimed to compare the retinal measures in three age subgroups, namely, younger, middle-aged, and older participants, distinguished from the samples of patients and from controls, and to verify the assumption that, in the clinical group, decreases in measures of MT, MV, GCC, and two types of RNFL will be more and more pronounced in successive age ranges. No such significant changes should occur in the control group. Additionally, we aimed to examine whether potential relationships between aging in schizophrenia and retinal measures were also present in every age range. Owing to the highly probable impact of disease on the retina, it seems plausible that the pattern of age and retina correlations in the clinical group will be different than that in controls. It was also expected that, in the schizophrenia sample, retinal variables will be significantly correlated with selected age-dependent cognitive functions based on information processing speed (19); concurrently, there will be no relationship with demographic characteristics and cognitive features that are typically age-independent, such as education and verbal knowledge.

## Methods

### Participants

Patients diagnosed with schizophrenia (F20.x, SCH group) according to the tenth revision of the International Classification of Disease [ICD-10, (20)] criteria were recruited from the Academic Psychiatry Clinics associated with the Medical University of Lublin. All individuals had to fall within the age range of 21–65 years and had to have at least 12 years of education. Subjects from the clinical group were excluded if they had a serious neurological or medical condition or a history of psychoactive substance (excluding nicotine) addiction in the past 6 months. All patients were treated with antipsychotic medications. Daily dosages were converted into risperidone equivalents (21). The assessment took place during psychiatric hospitalization, after a minimum of 4 weeks of treatment with antipsychotic drugs, after which functional improvement was achieved, enabling patients to participate in the study. Patients' clinical characteristics were reconstructed on the basis of the available medical records. Healthy controls matched for age, gender, and education were recruited from among those reporting for checkups to non-public healthcare facilities in the Lubelskie Voivodeship. All participants gave their written informed consent after receiving detailed information. The study was conducted following the Declaration of Helsinki and received written approval from the Bioethics Committee of the Medical University of Lublin (KE-0254/248/2020).

Regarding all participants, the following exclusion criteria were implemented: any previously diagnosed ophthalmological disorders (e.g., glaucoma, macular degeneration, and diabetic retinopathy), diabetes mellitus, non-treated arterial hypertension (if treated, BP should be > 140/90 mm Hg during an evaluation), obesity (BMI above 30), history of ocular trauma, ocular surgery, eye refraction above ± 5 dpt, glaucoma suspect (DDLS ≥ 6), dementia, and any relevant, concomitant psychiatric disorder (patients with F25.x were excluded from the study). Ophthalmological abnormalities were detected by a certified ophthalmologist. Interviews and ophthalmologic and cognitive evaluations were conducted on the same day. The psychiatric assessment took place within the 1-week time of the OCT test.

### Retinal morphology assessment: OCT

**Figure 1:**
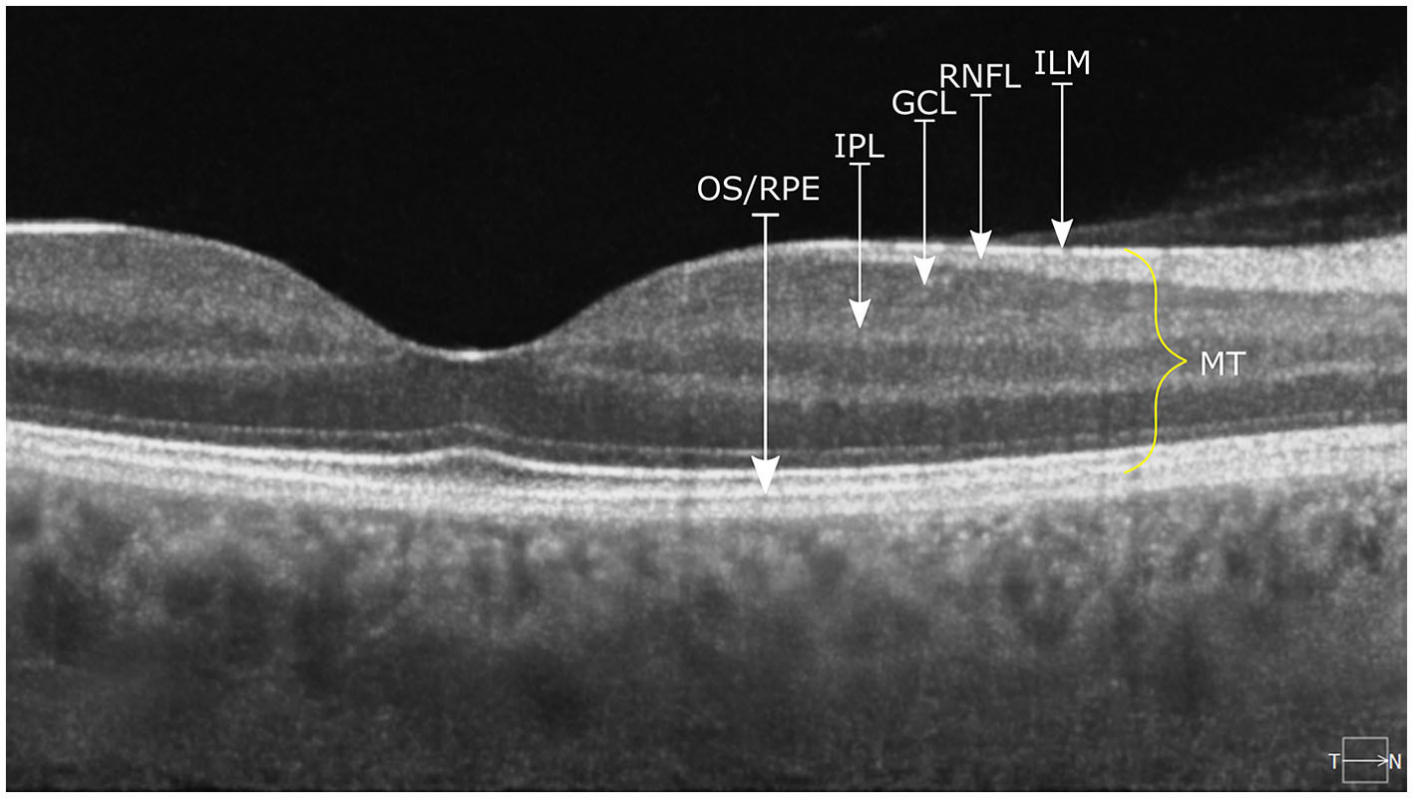
The OCT measurement of the macular layers, including RNFL and GCC. The picture was taken using OPTOPOL COPERNICUS REVO^®^ Spectral Domain OCT device, and it shows the retina of a healthy individual. ILM, internal limiting membrane; GCL, ganglion cell layer; IPL, inner plexiform layer; OS/RPE, outer segments/retinal pigment epithelium.

All participants underwent retinal imaging using the OPTOPOL COPERNICUS REVO^®^ Spectral Domain – OCT (OPTOPOL Technology, Zawiercie, Poland) with an upgraded scanning speed of up to 80, 000 measurements/s. The Spectral Domain – OCT is characterized by a SLED light source operating on a wavelength of 830 nm, an axial resolution of 2.6 μm, a transverse resolution of 12 μm, and a scan depth of 2.4 mm. All the results were processed with built-in OPTOPOL SOCT 11.0.7 software utilizing automatic segmentation of individual retinal layers with the manual correction of an experienced ophthalmologist. Only high-quality images were accepted (QI ≥7) for further analyses (Figure 1).

Measures of MV and MT were subdivided according to the ETDRS grid for both eyes. ETDRS grid divides the macula into three circles: central circle (1 mm), inner circle (3 mm), and outer circle (6 mm). The inner and outer circles could be additionally divided into four quadrants, namely, inferior, superior, nasal, and temporal. Measures of GCC (mRNFL+GCL+IPL) were subdivided into six segments, namely, superior, inferior, nasal superior, nasal inferior, temporal superior, and temporal inferior.

Images were acquired using a protocol consisting of 640 A-Scans and 85 B-scans in a 7 × 7 mm area centered in the fovea. MT and MV were measured from ILM to OS/RPE border. Peripapillary RNFL measures were obtained with a protocol of 512 A-scans and 112 B-scans in a 6 × 6 mm area centered in the optic disk. The measurements of RNFL were taken from the ring area of 2.4 mm diameter and 0.4 mm thickness from the optic disk. This area was divided into four quadrants, namely, inferior, superior, nasal, and temporal.

### Psychiatric and cognitive assessment

Schizophrenia psychopathology was evaluated using the Positive and Negative Syndrome Scale (PANSS). The PANSS employs a semi-structured interview assessing three domains, namely, Positive Symptoms Scale (7 items), Negative Symptoms Scale (7 items), and the General Psychopathology Scale (16 items) (22).

The overall level of cognitive functioning was assessed with the application of the ACE-III Scale (23). The cutoff point excluding dementia was set at 70 points (24). A vocabulary test from the WAIS-R(PL) Scale (25) was used to measure word knowledge and linguistic concept formation ability. Two indicators of cognitive functions dependent on performance speed were used: a Trail Making Test (TMT (26)) measuring mainly attention and psychomotor speed (part A) and mental flexibility (part B). The main outputs of TMT are time of performance and a number of errors informing about execution accuracy. In addition, to use a task with a time limit, but not dependent on the visual modality, the verbal fluency test in the phoneme version was applied. The subjects were asked to give as many unique words as possible for 60 s, beginning with a given letter (K, A, and T were used). The given words cannot be proper names and could not be repeated within one trial. The test result was the average number of unique words given in three attempts and the number of errors. Errors included perseveration and words that did not follow the instructions (27).

### Statistical analysis

Due to the results analysis plan containing many multivariate methods and the assessment of complex within- and between-group effects, all statistical analyses included the averaged parameters of the OCT results initially measured in separated quadrants. The raw values of MT, MV, mRNFL, pRNFL, and GCC were z-standardized to obtain outcomes adhering to the normal distribution. To verify whether retinal age-related trajectories were significantly different in groups of patients and controls, a two-way ANOVA was implemented, with diagnosis (HC vs. SCH) as the first factor and age range (younger, middle-aged, and older) as the second factor. The significance level was set at a *p-*value of <0.05, but it was adjusted to the impact of multiple comparisons by applying Bonferroni correction. If the age subgroups among the SCH sample differed significantly regarding OCT outcomes, an ANCOVA was used to test the potential impact of antipsychotic doses on these effects. For all ANOVAs, partial eta squared ($\eta_{p}^{2}$) was an effect size indicator. Pearson's correlation coefficient (*r*) was applied to assess relationships between studied variables, with the statistical significance level corrected for the number of performed analyses. If the correlations were found to be significant also after adjusting for multiple testing, the relationship was validated using a linear regression analysis including the risperidone equivalent as a controlled variable. In the Results section, tables contain raw metrics to show the values typical for the studied groups.

## Results

### Demographic and clinical characteristics of patients' and controls' age subgroups

Considering all previously described inclusion and exclusion criteria and the quality of OCT recordings, data from 60 SCH patients and 61 healthy controls (HC) have been ultimately analyzed. Taking into account participants' age distributions, each sample was divided into three subgroups. The younger (Y) participants were aged between 20 and 31 years, the middle-aged (M) participants were aged between 32 and 45 years, and the older (O) participants were aged between 46 and 65 years. Such age ranges enabled distinguishing subgroups with relatively similar participant numbers.

As shown in Table 1, the age subgroups in SCH and HC samples did not differ significantly regarding sex and years of education; in the HC sample, the age subgroups were also similar regarding the overall score on the ACE-III Scale; and in the SCH sample, the older ones scored significantly worse than the younger ones (*p* < 0.001). As for the SCH sample, the duration of untreated psychosis, number of hospitalizations, and the score in the PANSS subscale of Positive Symptoms did not differ in the subgroups; however, scores of Negative Symptoms (N) and General Pathophysiology Scale (G) subscales were significantly higher in the O or M subgroup compared with Y. Middle-aged patients were also treated with significantly higher doses of antipsychotics compared to the older ones. The SCH subgroups differed significantly regarding performance time of the Trail Making Test parts A and B and with reference to the verbal fluency test outcomes (Supplementary Table S1).

**Table 1:** Demographic characteristics of the study groups divided into three age intervals.

		**Younger (20–31 years) n, M (SD)**	**Middle-aged (32–45 years) n, M (SD)**	**Older (46–65 years) n, M (SD)**	**F/H**	***p***	*** $\eta_{p}^{2}$ ***	***Post-hoc***
Age	HC	*n =* 19/28.00 (3.91)	*n =* 23/42.50 (3.36)	*n =* 19/57.22 (2.42)	63.563	<0.001	0.69	Y <M <O
	SCH	*n =* 20/25.50 (3.67)	*n =* 21/38.35 (6.24)	*n =* 19/60.11 (5.24)	51.309	<0.001	0.65	Y <M <O
Education	HC	14.84 (2.31)	13.87 (2.47)	13.11 (2.13)	2.571	0.085	0.08	-
	SCH	12.65 (2.18)	13.19 (2.42)	13.84 (3.48)	1.264	0.29	0.04	-
Sex	HC	12	14	10	2.152	0.34	0.06	-
*n* men	SCH	11	13	10	0.525	0.768	0.02	-
ACE-III	HC	94.78 (4.44)	93.71 (4.14)	90.55 (5.67)	2.89	0.063	0.09	-
	SCH	86.20 (6.84)	80.90 (10.99)	71.72 (6.10)	14.173	<0.001	0.33	Y > O

### Comparisons of retinal age-related changes in SCH and HC samples: two-way ANOVA effects

Table 2 consists of all evaluated retinal measures (MT, MV, mRNFL, pRNFL, GCC) in the age subgroups with effects of age (Y vs. M vs. O) and effects of diagnosis (HC vs. SCH). According to the presented results, none of the interaction effects turned out to be significant.

**Table 2:** Effects of two-way ANOVA (diagnosis × age subgroups) for the retinal parameters.

			**Younger M (SD)**	**Middle-aged M (SD)**	**Older M (SD)**	**Interactioneffect F**	**p**	*** $\eta_{p}^{2}$ ***	**Age effect F**	**p**	*** $\eta_{p}^{2}$ ***	**Groupeffect F **	**p**	*** $\eta_{p}^{2}$ ***	**Group effect *Post-hoc***	Interaction effect *Post*−hoc^*^
R	Macular thickness	HC	284.32 (12.80)	285.44 (11.31)	278.94 (11.87)	1.390	0.252	0.02	3.230	0.043	0.05	26.550	<0.001	0.19	HC > SCH	HCm > SCHm HCo > SCHo SCHy > SCHo
		SCH	277.26 (7.36)	269.79 (11.58)	269.45 (9.41)											
L	Macular thickness	HC	283.88 (13.06)	284.89 (12.10)	277.50 (11.64)	0.550	0.578	0.01	2.500	0.086	0.04	17.770	<0.001	0.13	HC > SCH	HCm > SCHm
		SCH	277.37 (6.90)	270.04 (13.84)	271.85 (13.90)											
R	Macular volume	HC	7.98 (0.35)	8.02 (0.32)	7.88 (0.33)	1.552	0.222	0.02	3.610	0.030	0.06	27.788	<0.001	0.19	HC > SCH	HCm > SCHm HCo > SCHo SCHy > SCHo
		SCH	7.83 (0.21)	7.62 (0.32)	7.61 (0.26)											
L	Macular volume	HC	8.03 (0.36)	7.99 (0.33)	7.84 (0.33)	0.580	0.562	0.01	2.500	0.088	0.04	18.080	<0.001	0.13	HC > SCH	HCm > SCHm
		SCH	7.84 (0.19)	7.63 (0.38)	7.71 (0.39)											
R	Macular RNFL	HC	27.82 (1.63)	28.08 (2.45)	26.93 (2.14)	1.910	0.152	0.03	0.120	0.887	0.01	0.130	0.722	0.01	-	-
		SCH	28.61 (2.05)	27.78 (2.67)	27.43 (3.47)											
L	Macular RNFL	HC	28.12 (1.87)	28.71 (2.41)	26.68 (2.08)	1.770	0.174	0.03	1.880	0.156	0.03	0.911	0.342	0.01	-	-
		SCH	27.68 (1.88)	27.78 (2.67)	27.69 (2.25)											
R	Peripheral RNFL	HC	115.91 (1.87)	116.72 (7.48)	114.41 (4.68)	0.400	0.960	<0.01	0.919	0.402	0.01	19.867	<0.001	0.14	HC > SCH	-
		SCH	109.65 (15.21)	105.26 (12.86)	104.38 (9.43)											
L	Peripheral RNFL	HC	113.26 (2.29)	117.77 (13.42)	111.91 (7.31)	0.244	0.783	<0.01	1.030	0.360	0.09	21.936	<0.001	0.16	HC > SCH	-
		SCH	108.00 (12.26)	102.80 (14.05)	102.04 (12.68)											
R	GCC	HC	119.26 (7.67)	118.33 (8.62)	115.00 (9.21)	0.040	0.937	<0.01	3.130	0.047	0.05	9.480	0.002	0.08	HC > SCH	-
		SCH	114.45 (3.39)	114.45 (8.05)	110.35 (8.06)											
L	GCC	HC	119.79 (7.51)	118.21 (8.68)	114.17 (9.00)	0.040	0.964	<0.01	3.900	0.022	0.06	10.260	0.001	0.08	HC > SCH	-
		SCH	114.74 (3.33)	113.05 (8.74)	109.94 (9.13)											

^*^p < 0.003 Bonferroni-corrected level of statistical significance for interaction effects. Analyzing *post-hoc* comparisons, we aimed to detect differences between the comparable HC and SCH subgroups of the same age range, for example, the difference between patients and controls aged 21–31 years. These differences were considered significant only if the p-level reached statistical significance corrected for multiple testing.

**Figure 2:**
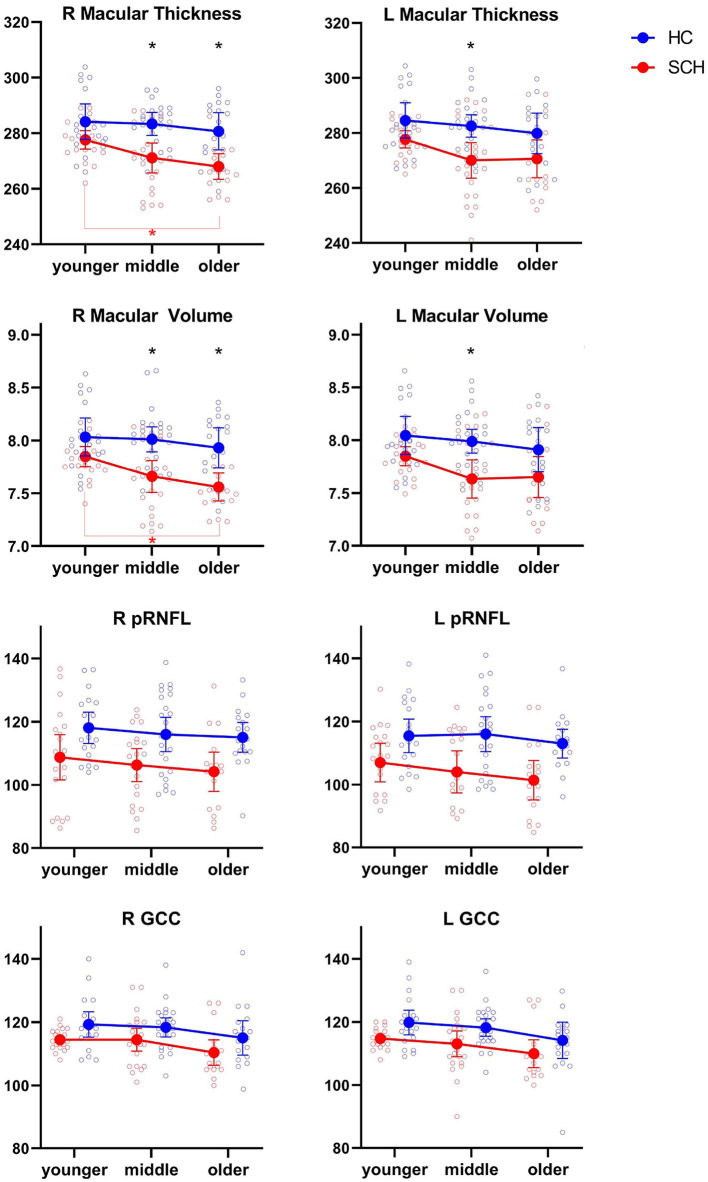
Scatter plots of two-way ANOVA for MT, MV, pRNFL, and GCC in the HC and SCH age subgroups. Vertical bars represent 0.95 confidence intervals. Black asterisks at the top of the figures indicate significant differences between the age subgroups of patients and controls. The red line and asterisk at the bottom indicate significant differences between the age subgroups of patients.

For the right MT, the age effect was non-significant concerning the adjusted level of statistical significance (*p* > 0.016). The group effect was significant (HC > SCH, *p* < 0.001), and as shown via the *post-hoc* analysis, it originated from the difference between the middle-aged and older subgroups of controls and patients. Within the SCH sample, right MT was significantly decreased in older patients compared with younger ones, and this difference remained significant also after controlling for risperidone equivalents (*p* = 0.004). The diagnosis effect was significant also regarding left MT (*p* < 0.001) and resulted from significant differences between middle-aged patients and controls. As for the right MV, a significant effect of diagnosis (*p* < 0.001) was driven by the difference between middle-aged and older patients and controls. Younger patients had significantly greater right MV than the older ones, and again, this difference remained significant also after controlling for risperidone equivalents (*p* = 0.002). Regarding left MV, the effect of diagnosis was significant (*p* < 0.001) and again resulted from the differences between middle-aged patients and controls. No significant effects were noticed regarding right and left mRNFL. Right peripapillary RNFL was significantly smaller in patients compared with controls (*p* < 0.001); however, no *post-hoc* tests reached the adjusted level of significance. Regarding left pRNFL, the results were similar, with a significant diagnosis effect (*p* < 0.001). Significant diagnosis effects concerned also right and left GCC (both *p* < 0.002), but without significant interaction *post-hoc* tests. Figure 2 represents the results of the analyzed variables in the HC and SCH age subgroups for MT, MV, pRNFL, and GCC in the right and left eyes.

### Associations between age and retinal parameters in the studied groups

The next step of the analysis involved verifying the associations between age and retinal parameters in the SCH and HC groups. To reduce the number of variables analyzed at once, the retinal measures for both eyes were averaged. No significant correlations were noted in the HC sample. In the complete SCH sample, the following retinal parameters correlated significantly with age: MT (*r* = −0.47, *p* < 0.0001), MV (*r* = −0.49, *p* < 0.0001), and GCC (*r* = −0.29, *p* = 0.023, this *p*-level, however, does not survive the correction for multiple analyses). The correlations between age and pRNFL and between mRNFL and age were non-significant (*p* > 0.1). For MT and MV as dependent variables, a linear regression analysis was performed to verify whether correlations with patients' age remained significant also when controlling for the risperidone equivalent (RE). Age was not correlated with risperidone equivalents. Both regression models were statistically significant (Supplementary Table S2). Despite the relatively weak correlation between GCC and age, a regression analysis was carried out to determine a possible relationship also after controlling for risperidone equivalent. The regression model turned out to be statistically significant (*p* = 0.022), although the range of explanation of the dependent variable was small (corrected *R*^2^ = 0.09, Supplementary Table S2). Considering the significant results of the regression analysis for age, MT, MV, and GCC, additional correlational analyzes were performed in the age subgroups of patients. In the middle-aged subgroup, age correlated with MT (*r* = −0.43, *p* = 0.045) and with MV (*r* = −0.45, *p* = 0.033); however, the significance level of these correlations did not survive the multiple testing correction.

### Relationships between retinal parameters and selected cognitive functions in the SCH sample

In the whole SCH group, there were no significant correlations between MT, MV, pRNFL, GCC, years of education, and vocabulary (all *p* > 0.09). After controlling for multiple testing, the performance time of the Trail Making Test part A correlated significantly with MV: *r* = −0.52, *p* < 0.001, GCC: *r* = −0.50, *p* < 0.001. A number of errors committed in this task correlated with MV: *r* = −0.31, *p* = 0.033; however, the level of statistical significance did not survive the correction for multiple testing. The performance time of TMT part B correlated significantly, also after controlling for multiple testing with GCC: *r* = −0.43, *p* = 0.003. The number of errors committed during TMT part B execution correlated significantly with GCC: *r* = −0.54, *p* < 0.001. Regression analyses including the above cognitive measures, risperidone equivalents, and identified OCT correlates turned out to be significant (*p* < 0.001, Supplementary Table S3), with one exception: the relationship between performance time of TMT part B and GCC ceased to be statistically significant after adding the risperidone equivalent as a control variable.

Verbal fluency output correlated significantly with GCC: *r* = 0.44, *p* = 0.002. This relationship remained significant also in regression analysis, with risperidone equivalent as a controlled variable (*p* < 0.001, Supplementary Table S3). The number of errors committed in the fluency test did not correlate significantly with any of the OCT results in the group of patients (*p* > 0.055). No significant correlations between analyzed cognitive and retinal measures were noted in the SCH age subgroups.

## Discussion

Our study aimed to verify whether retinal parameters such as macular thickness and volume, RNFL, and GCC measured in the schizophrenia sample exhibit significantly different age-related changes than in the control group. We expected a significant retinal thinning ongoing with the age of patients; additionally, we aimed to check whether the associations between age and OCT outcomes will be noticeable through all age ranges. According to the accelerated aging hypothesis (18), we also aimed to confirm that the retinal variables correlated significantly with those cognitive functions, which showed a significant age-related decline in the patients' group.

It has been documented that, in normal aging, a slight but tangible decrease in some retinal layers occurs (28, 29). Owing to the undoubted impact of schizophrenia on the nervous system, including the retina, we expected to observe a marked age-related decrease in retinal layer thickness in schizophrenia patients, which is not noticeable in the control group. Contrary to our expectations, none of the interaction effects (diagnosis × age subgroups) reached the level of statistical significance. Only *post-hoc* tests showed that macular thickness and volume in schizophrenia patients undergo a partially different range of changes than in controls. Significant diagnosis effect regarding macular parameters was mainly due to the differences between middle-aged patients and controls (aged 32–45 years). Considering the above, with some caution, it can be stated that, among schizophrenia patients, macular parameters deteriorate in an accelerating manner. A significant age-related decrease in the MT and MV was noted in the patients' group, and the lack of such significant changes in RFNL and GCC may indicate that accelerated aging applies mainly to the inner nuclear layer (INL), the outer plexiform layer (OPL), and the outer nuclear layer (ONL). Taking into account the data of evaluating the relationships between retinal layers and age in healthy subjects (30), our results regarding schizophrenia may, even more, indicate pathological changes in macular layers, because according to normative data, INL, OPL, and ONL do not express significant age-related decline.

Macular measures were significantly associated with age only in the patients' group, after controlling for antipsychotic dosage. Analyses in the age subgroups indicated that the mentioned decrease occurred mainly between 32 and 45 years of age. It is worth noting that the subgroup of younger patients (aged 20–31 years) did not differ in any of the OCT outcomes from their healthy peers, and no correlations with age and retinal variables were found among them. In this context, the lack of age vs. retina correlations in younger and older patients indicates that age-related retinal shrinkage in schizophrenia occurs mainly over a relatively narrow lifespan interval. Note that, from the beginning of patients' third decade of life, or by reaching approximately 10 years of the illness duration, a significant thinning acceleration might be observed through the next 10–15 years, which later slows down. To the best of our knowledge, this aspect has not been detected to date. Discussed patterns of retinal atrophy progression may explain previous findings according to which the greatest differences between patients and controls in retinal morphology occur when the patients with a longer duration of illness were enrolled (13, 14, 31). Studies referring to the accelerated brain aging hypothesis in schizophrenia not only corroborate the existence of the brain age gap (32, 33) but also have demonstrated that various types of brain tissues speed up aging at different disease stages. For example, according to Schnack et al.'s (34) study based on gray matter, accelerated aging was most pronounced at the disease onset and normalized about 5 years later, while Wang et al.' (35) study suggests that the white matter began to age faster starting only from the third decade of life. Considering the above, our outcomes regarding macular thickness and volume bear some similarity to the results of white matter aging in schizophrenia.

Despite numerous evidence confirming retinal abnormalities in schizophrenia (7, 9), only a few studies aimed to identify possible cognitive correlates of these alterations (36, 37). Referring to the accelerated aging hypothesis, we assumed that the OCT outcomes' decline in schizophrenia would be selectively related to those cognitive processes which, according to available literature, show an age-related weakening in the general population and reveal a significant decline with disease progression. This study our results fully confirmed these assumptions.

Summing up, we showed that, in schizophrenia patients, the retinal macula undergoes speeded atrophy from the third decade of life. It resembles the dynamics of white matter changes analyzed concerning the accelerated aging hypothesis (38). Curves indicating age-related modifications in other retinal structures were generally very similar in both groups, just with more pronounced thinning in patients' samples. Importantly, associations between the macula, GCC, and patients' age concerned only the middle-aged subgroup, which suggests that retinal abnormalities in schizophrenia do not increase linearly over the entire life. Particularly interesting is their absence in patients over 50 years of age/30 years of disease duration. Correlations between macular thickness and volume with speed-dependent cognitive functions, including verbal fluency, are in line with the accelerated aging approach; however, the overall scope of analyzed cognitive functions was limited. These associations need further exploration.

### Limitations

All performed analyses concerned only the averaged parameters of retinal layers' morphology; therefore, the obtained results may not reflect the potential changes occurring in individual retinal quadrants with the disease progress (39). As shown in Figure 2, macular thickness and volume decreased more prominently with age in the SCH groups compared with controls; however, no interaction effects reached the statistical significance level. These results might be conditioned by relatively small age subgroups, which probably increased the impact of morphological measures variability. The conclusions about the possible aging acceleration of macula in schizophrenia patients are not based on advanced computational methods applied in neuroimaging studies using MRI or DTI but rather a preliminary suggestion regarding the possibility of interpreting the obtained results. Considering the anatomical complexity of the retina (40, 41) it seems possible to apply machine learning to calculate retinal layers' age in the large research group, and such analysis would ultimately determine the validity of our hypothesis.

## Data availability statement

The raw data supporting the conclusions of this article will be made available by the authors, without undue reservation.

## Ethics statement

The studies involving human participants were reviewed and approved by the Bioethics Committee of the Medical University of Lublin. The patients/participants provided their written informed consent to participate in this study.

## Author contributions

AD and PK designed the study. AD and LD collected the samples. NK-P and MH performed the literature review, and conducted the analyses. PK, MH, and AD wrote the initial version of this manuscript. All authors contributed to the article and approved the submitted version.
