# Supplementary material for: Differentiation of the retinal morphology aging trajectories in schizophrenia and their associations with cognitive dysfunctions
Source: Front Psychiatry. 2023 Jul 19;14:1207608. doi: 10.3389/fpsyt.2023.1207608 (PMC10396397; doi:10.3389/fpsyt.2023.1207608)
Supplement: Supplementary file 1 [file Data_Sheet_1.docx]

SUPPLEMENTARY MATERIAL

Differentiation of the retinal morphology aging trajectories in schizophrenia

and their associations with cognitive dysfunctions

**Table 1S.** Comparison of clinical and cognitive characteristics in age-subgroups in the schizophrenia sample.

|  | Younger  M (SD) | Middle  M (SD) | Older  M (SD) | F | p | *η_p_^2^* | post hoc |
| --- | --- | --- | --- | --- | --- | --- | --- |
| DUP | 1.62 (1.77) | 2.21 (3.43) | 2.94 (2.98) | 0.885 | 0.418 | 0.03 | - |
| DUI | 5.53 (3.81) | 15.91 (8.33) | 31.47 (11.74) | 50.110 | < 0.001* | 0.54 | Y < M < O |
| Number of hosp. | 2.15 (1.41) | 7.31 (3.73) | 5.94 (6.63) | 3.091 | 0.053 | 0.05 | - |
| PANSS P | 16.00 (5.13) | 18.18 (4.71) | 19.94 (6.35) | 1.885 | 0.161 | 0.06 | - |
| PANSS N | 19.20 (6.87) | 21.77 (5.53) | 24.47 (5.06) | 6.802 | 0.002* | 0.19 | Y < O |
| PANSS G | 42.45 (7.49) | 40.18 (8.52) | 50.05 (9.13) | 8.131 | < 0.001* | 0.22 | M < O |
| PANSS total | 77.65 (17.40) | 80.13 (18.88) | 94.46 (15.06) | 6.344 | 0.003 | 0.18 |  |
| Typical / Atypical | 1/18 | 3/20 | 6/13 |  |  |  |  |
| Risperidone equivalent | 12.73 (6.11) | 14.73 (8.38) | 6.98 (7.25) | 6.795 | 0.002* | 0.19 | M > O |
| WAIS-R Vocabulary | 8.89 (4.64)^a^ | 8.29 (3.10) | 8.14 (3.88) | 1.086 | 0.378 | 0.04 | - |
| TMT part A perf. time | 44.66 (15.96) | 80.52 (59.81) | 148.40 (78.44) | 6.871 | 0.002* | 0.21 | Y < O |
| TMT part A errors | 0.16 (0.38) | 0.42 (0.62) | 1.00 (1.44) | 1.441 | 0.246 | 0.05 | - |
| TMT part B perf. time | 127.33 (59.81) | 203.30 (73.17) | 361.81 (152.81) | 9.744 | < 0.001* | 0.29 | Y < O |
| TMT part B errors | 0.61 (0.69) | 1.00 (1.83) | 2.36 (1.43) | 5.419 | 0.007 | 0.19 | - |
| Verbal fluency – mean number of words | 12.05 (3.10) | 11.70 (4.22) | 5.20 (2.58) | 23.559 | < 0.001* | 0.48 | Y, M > O |
| Verbal fluency – number of errors | 1.27 (2.02) | 1.28 (1.70) | 2.53 (3.50) | 1.443 | 0.245 | 0.05 | - |

***** *p* significant after Bonferroni correction for multiply comparisons

^a^ normalized results

DUP – duration of untreated psychosis (in moths)

DUI – duration of illness (in years)

Typical / Atypical antipsychotics

perf. time – time of performance in seconds

mean number of words from 3 trials

**Table 2S.** Results of the linear regression analyses with MT and MV as dependent variables, age and risperidone equivalents as predictors in the SCH group.

| Dependent variable | Predicates | β | St. error | t | p | R^2^ | Corr. R^2^ | ANOVA |
| --- | --- | --- | --- | --- | --- | --- | --- | --- |
| MT | Age  Risperidone equivalent | **-0.53**  **-0.33** | **0.13**  **0.13** | **-4.034**  **-2.469** | **0.001**  **0.016** | 0.23 | 0.20 | *F*(2, 57) = 9.810, *p* = 0.002, *η_p_^2^*= 0.21 |
| MV | Age  Risperidone equivalent | **-0.55**  -0.20 | **0.13**  0.13 | **-4.209**  -1.402 | **0.001**  0.162 | 0.24 | 0.21 | *F*(2, 57) = 11.492, *p* = 0.001*η_p_^2^*= 0.22 |
| GCC | Age  Risperidone equivalent | **-0.36**  -0.17 | **0.14**  0.14 | **-2.848**  -1.215 | **0.006**  0.229 | 0.12 | 0.09 | *F*(2, 57) = 4.056, *p* = 0.022*η_p_^2^*= 0.13 |

*Note*. Bold type indicates statistically significant predictors.

**Table 3S.** Results of the linear regression analyses with speed-related cognitive measures as dependent variables, and selected OCT outcomes and risperidone equivalents as predictors in the SCH group.

| Dependent variable | Predicates | β | St. error | t | p | R^2^ | Corr. R^2^ | ANOVA |
| --- | --- | --- | --- | --- | --- | --- | --- | --- |
| TMT part A performance time | MV  Risperidone equivalent | **-0.40**  **0.29** | **0.11**  **0.11** | **-3.336**  **-2.439** | **0.001**  **0.018** | 0.23 | 0.21 | *F*(2, 56) = 7.943, *p* < 0.001, *η_p_^2^*= 0.16 |
| TMT part A performance time | GCC  Risperidone equivalent | **-0.42**  0.23 | **0.12**  0.12 | **-3.447**  -1.901 | **0.001**  0.062 | 0.24 | 0.22 | *F*(2, 56) = 8.347, *p* < 0.001, *η_p_^2^*= 0.18 |
| TMT part B performance time | GCC  Risperidone equivalent | -0.25  **0.35** | 0.13  **0.13** | -1.929  **-2.613** | 0.059  **0.012** | 0.20 | 0.16 | *F*(2, 56) = 5,826, *p* = 0.005, *η_p_^2^*= 0.14 |
| TMT part B number of errors | GCC  Risperidone equivalent | **-0.43**  **0.39** | **0.11**  **0.11** | **-3.595**  **-3.276** | **0.001**  **0.002** | 0.36 | 0.33 | *F*(2, 56) = 13.122, *p* < 0.001, *η_p_^2^*= 0.28 |
| Letter fluency –mean words number | GCC  Risperidone equivalent | **0.28**  **0.42** | **0.12**  **0.12** | **2.298**  **3.599** | **0.030**  **0.001** | 0.26 | 0.23 | *F*(2, 56) = 9.117, *p* < 0.001, *η_p_^2^*= 0.20 |

*Note*. Bold type indicates statistically significant predictors.

**Figure 1S.** Diagram showing the main relationships between the variables studied in the SCH group.

pRNFL

+ Risperidone Eq.

+ Risperidone Eq.

+ Risperidone Eq.

+ Risp. Eq.

AGE

mRNFL

MT

MV

GCC

*Note.* Black arrows – significant predictions, light gray arrows - verified but ultimately statistically non-significant relationships. + Risperidone Eq. – relationships with significant impact of antipsychotics.
